# Application of contrast-enhanced ultrasound in the diagnosis of post-transplant lymphoproliferative disease after hematopoietic stem cell transplantation: A case report

**DOI:** 10.1097/MD.0000000000024047

**Published:** 2021-01-15

**Authors:** Weinan Chen, Jianchun Li, Xiaoming Fan, Yanming Zhang, Li Wang, Yang Liu, Ailin Cui, Ligang Wang

**Affiliations:** Zhejiang Provincial People's Hospital, People's Hospital of Hangzhou Medical College, Hangzhou, Zhejiang, China.

**Keywords:** contrast-enhanced ultrasound, hematopoietic stem cell transplantation, post-transplant lymphoproliferative disease

## Abstract

**Introduction:**

Post-transplant lymphoproliferative disease (PTLD) is a series of proliferative diseases of the lymphatic system. Among patients receiving hematopoietic stem cell transplantation (HSCT), PTLD is a prevalent complication that severely affects rates of survival. Ultrasound plays an essential role in the early diagnosis of PTLD. Contrast-enhanced ultrasonography (CEUS) and CEUS-guided biopsy are critical procedures for tumor diagnosis.

**Patient concerns:**

Herein, we report the case of a 40-year-old male patient with acute lymphoblastic leukemia who received HSCT more than 1 year ago. Sonography revealed a small hypoechoic nodule in the liver four months after HSCT. Eight months after HSCT, larger and more nodules were observed via ultrasound; CT was used to identify the lesions.

**Diagnoses:**

CEUS and CEUS-guided biopsy were performed, and the pathological diagnosis was PTLD.

**Interventions:**

The final clinical diagnosis was PTLD, and cyclophosphamide, epirubicin, and dexamethasone were administered as chemotherapy.

**Outcomes:**

The patient was discharged after his condition improved.

**Conclusion:**

Ultrasound can be used to effectively detect lesions of PTLD early after HSCT. Furthermore, CEUS and CEUS-guided biopsy were effective for early confirmatory diagnoses of PTLD after HSCT.

## 1 Introduction

Post-transplant lymphoproliferative disease (PTLD) is characterized by abnormal lymphoid proliferation, from benign to malignant, caused by immunosuppression following solid organ or hematopoietic stem cell transplantation (HSCT).^[1]^ Early diagnosis of PTLD is critical for improving patient prognosis and preventing further development of malignant lymphoma^[2]^ PTLD can be diagnosed using different imaging methods including ultrasonography (US), computed tomography (CT), contrast-enhanced computed tomography (CE-CT), magnetic resonance imaging, and position emission tomography/computed tomography. However, position emission tomography/computed tomography is radioactive and therefore, inappropriate when multiple examinations are indicated.^[3]^ The use of CE-CT and magnetic resonance imaging is allergic and nephrotoxic potential of the employed contrast materials.^[4]^ In addition, patients with leukemia usually have renal impairment due to chemotherapeutic drugs.^[5]^ Meanwhile, ultrasound is the preferred surveillance strategy, as it is an accessible and inexpensive imaging method for routine monitoring of post-transplant patients.^[6]^ In addition, contrast-enhanced ultrasonography (CEUS) is less invasive, has no associated nephrotoxicity, allows for real-time observation, and has fewer side effects. It can also be used for the accurate assessment of tumor vascularity, tumor location, and other defining features that aid in subsequent puncture operations.^[7]^ Therefore, ultrasound has gradually become the preferred inspection method for patients following transplantation.^[8]^ Herein, we report a case involving a 40-year old man with hypoechoic nodules in the liver associated with PTLD. We provide detailed information and characteristics of CEUS of the tumor and describe the process of ultrasound-guided biopsy. There have been no noted previous reports on CEUS presentation of adult intrahepatic PTLD after HSCT.

## 2 Case presentation

**Figure 1:**
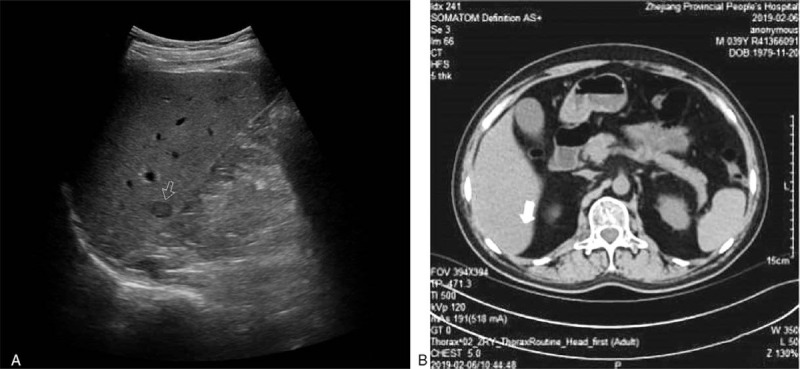
The same level of ultrasound and computed tomography (CT) images. (A) Ultrasound revealed a 1.5 cm × 0.9 cm hypoechoic nodule. (B) At the same level, computed tomography (CT) did not detect any solid lesion.

**Figure 2:**
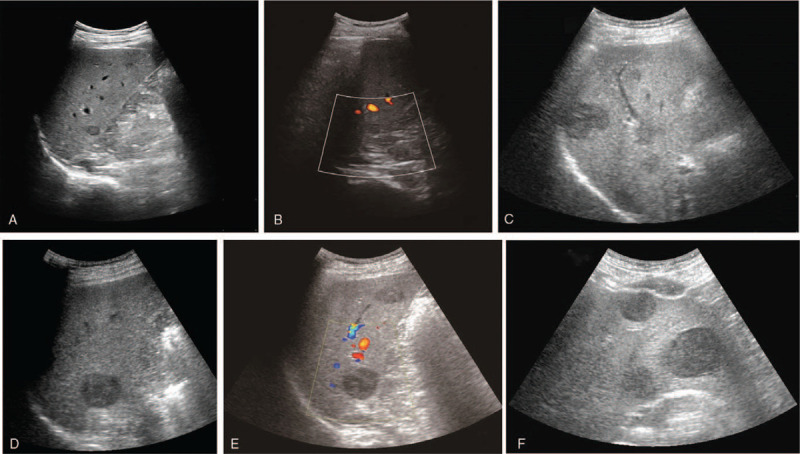
Sonographic images of nodules changing from less to more. (A) The nodule first identified by ultrasound. (B) No obvious blood flow signal was found in the nodule first identified. (D) The nodule was initially found enlarged. (E) No obvious blood flow signal was found in the nodule was initially found enlarged. (C,F) Sonographic images of a single nodule developing into multiple nodule.

A 40-year-old male patient was admitted to our hospital with recurrent gingival bleeding and was diagnosed with acute lymphoblastic leukemia 15 months before the study. The patient underwent allogeneic HSCT 7 months after diagnosis. During this time, the patient was noted to be in good health. Chemotherapy and supportive treatment were maintained after the transplant. Four months after transplantation, hypoechoic nodules were detected in the liver and spleen via ultrasound (Fig. 1A), but these were not detected on CT (Fig. 1B). No intrahepatic lesion was found on PET/CT and multiple ultrasound examinations before transport. US suggested a possible diagnosis of a malignant lesion, similar to CEUS, but it was not adopted. Laboratory indicators were positive for Epstein-Barr virus (EBV) and cytomegalovirus, and bilirubin and creatinine were also elevated. Four more months passed, and sonography identified more and larger intrahepatic nodules (Fig. 2A–F). Nodules in the liver were larger than originally observed (Fig. 2D), but hypoechoic nodules in the spleen disappeared, as confirmed by the CT scan results. Ultrasound results were similar to those from the first diagnosis. The patient was referred to our department for further evaluation and diagnosis of the tumor via CEUS and ultrasound-guided puncture.

**Figure 3:**
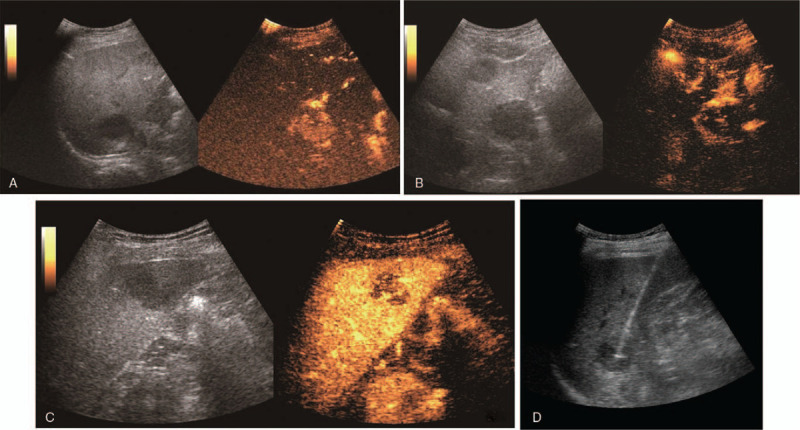
Contrast-enhanced ultrasonography and ultrasound-guided puncture of the tubercle. (A) Contrast-enhanced ultrasonography of nodule 1. (B) Contrast-enhanced ultrasonography of nodule 2. (C) Contrast-enhanced ultrasonography of nodule 3. (D) Ultrasound-guided puncture image.

**Figure 4:**
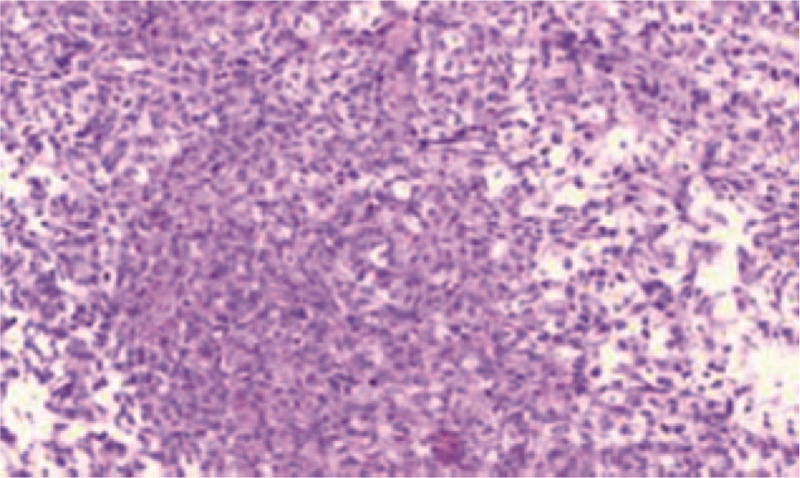
Pathological results: diffuse proliferation of lymphoid cells was observed in the hepatic portal area and between hepatic sinuses, it is suitable for PTLD, lymphoma stage, and B cell lymphoma after transplantation.

US was performed using the LOGIQ E9 (GE, America) equipped with a convex transducer (frequency range 1–6 MHZ). Two-dimensional ultrasound showed multiple low-echo nodules in the liver with clear boundaries, uneven internal echoes, and surrounding hypoechoic halos. Color Doppler ultrasound showed no obvious blood flow signal in the nodules (Fig. 2B,E). We performed CEUS on 3 hepatic nodules (Fig. 3A–C): the largest nodule in the left lobe of the liver (nodule 1), the nodule in segment VI of the liver was enlarged and in the original position (nodule 2), and one nodule in the right lobe of the liver (nodule 3). Then, a bolus of 1.2 mL of Sonovue (Bracco, Milan, Italy) was administered intravenously, and each node was flushed using 5.0 mL of 0.9% saline. CEUS of nodule 1 (approximately 4.4 × 3.4 cm) (Fig. 3A): The annular enhancement of arterial stage was 12 s earlier than peripheral parenchymal, peak intensity was similar to peripheral tissue, the funicular and lamellar enhancement areas were visible internally, no enhancement zone persisted at all times, and the contrast agent was removed from the lesion at 134 s during the delay period. CEUS of nodule 2 (approximately 3.2 × 2.7 cm) (Fig. 3B): The nodule was observed with peripheral annular enhanced at 15 seconds of the arterial stage, cable-like reinforcement was observed internally, and the contrast agent was removed in the delay period at 144 seconds. CEUS of nodule 3 (approximately 2.5 × 2.3 cm) (Fig. 3C): A completely homogeneous enhancement of the arterial stage was observed 14 s earlier than the surrounding tissues, and the peak intensity was similar at the periphery. After 32 seconds, the lesion became hypo-enhanced at the portal venous phase. The characteristics of CEUS suggested the possibility of malignant lesions. Next, we performed an ultrasound-guided biopsy of nodule 2 (Fig. 3D) because it was the first isolated hepatic nodule identified by ultrasound. Several site tissues were punctured successfully using a BARD automatic biopsy gun with an 18G percutaneous core needle biopsy guided by CEUS. Pathological results were consistent with PTLD (Fig. 4), lymphoma stage, and B-cell lymphoma. Immunohistochemical staining showed positive results for CD19, CD79a, CD34, CD20, and Ki67. The results of molecular monitoring were A2-1: EBER(+). The final clinical diagnosis was PTLD, and cyclophosphamide, epirubicin, and dexamethasone were administered for chemotherapy. The patient was discharged after his condition improved. The report was approved by the Ethics Review Board of Zhejiang Provincial People's Hospital. The patient provided informed consent for publication of the case.

## 3 Discussion and conclusions

PTLD is a serious complication of high heterogeneity following solid organ and HSCT, and it has high rates of morbidity and mortality.^[9,10–14]^ PTLD usually occurs within 6 to 12 months after HSCT, before the reconstruction of EBV-specific cytotoxic T-cell immunity.^[10,11,15,16]^ In allogeneic HSCT, previous studies demonstrated that the incidence rate of PTLD following allogeneic HSCT varied greatly from 1.0% to 17%. It also varied with the stem cell source, degree of HLA mismatch, and patient characteristics. Conditioning eigenfactors can contribute to the development of PTLD, including the degree of human leukocyte antigen mismatch along with aggressive T-cell depletion methods.^[17]^ Prompt diagnosis is crucial to prevent the development of malignant lymphoma.

Multiple imaging diagnostic methods are essential tools for early diagnosis and staging of lesions, and ultrasonic examination is a common surveillance strategy for patients after transplantation.^[18]^

In our case, the intrahepatic lesion was detected early by ultrasound, and CT was not possible. Differences between US and CT may be attributed to the patient receiving plain CT instead of enhanced CT. Plain CT is best for medium density tissues and cannot differentiate between normal and diseased tissues. Because of the patient's abnormal liver and renal function, enhanced CT was not the preferred method of examination. Furthermore, the tumor was small and located in segment VI of the right posterior lobe of the liver in the low position; hence, plain CT might have omitted it. Although the patient did not undergo enhanced CT, we considered CEUS to be the more suitable modality for routine examination and diagnosis of the tumor. First, two-dimensional ultrasound showed no obvious blood flow signal in the lesion. Ultrasound contrast media flow of blood has a better correlation with tumor microvascular density. Therefore, CEUS is more sensitive than CE-CT for detecting low vascular lesions.^[19]^ Second, CEUS can more efficiently identify tumor characteristics, vascular distribution, and dynamic relationships with surrounding tissues in real time. It is also safer for patients with abnormal liver and kidney function. Third, there were necrotic areas within the partial nodules in our case. CEUS can be used to distinguish between enhanced activity areas and nonenhanced necrosis. Ultrasound-guided puncture can reduce the acquisition of necrotic tissue and obtain greater value pathological tissue.^[20]^ Furthermore, a study showed that, as a radiation-free and widely used imaging method, US is reliable for the detection and exclusion of abdominal or soft tissue lymphoma in children with PTLD, while CT may be reserved for supplementary imaging in cases with incomplete or equivocal findings on US.^[8]^ In addition, false-negative results of PET/CT mainly occur in areas with high physiological background activity and early PTLD lesions.^[9]^ Therefore, US and CEUS examinations have significant practical value in this case.

**Figure 5:**
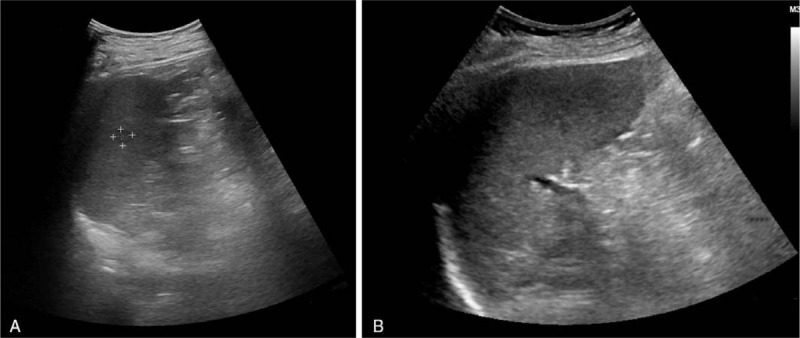
Contrast of spleen ultrasound images. (A) Ultrasound of intrasplenic nodule. (B) Splenomegaly ultrasonography.

It is worth noting the disappearance of the splenic nodule (Fig. 5A,B). Because of the increased creatinine in the patient, cyclosporine was discontinuation, methylprednisolone and tacrolimus were administered. A previous case reported that treatment with methylprednisolone and tacrolimus after umbilical cord blood transplantation could prevent graft-vs-host disease.^[21]^ Thus, we suggest that the intrasplenic nodule was not of the same nature as the intrahepatic nodule and might be related to a manifestation of graft-vs-host disease. Thus, changes in drugs administered may be responsible for the disappearance of the nodule. We hope that future investigations will elucidate these causes.

To the best of our knowledge, the use of US and CEUS for the detection of intrahepatic PTLD after HSCT has not been systematically reported. In our case, two-dimensional ultrasound revealed multiple hypoechoic nodules in the liver with clear boundaries, neat contours, uneven internal echoes, and no obvious blood flow signals. CEUS showed that the foci with necrotic areas presented rapid annular enhancement in the arterial phase; while in the initial primary foci with nonnecrotic areas, there was rapid global enhancement. Low enhancement was observed in both the venous and parenchymal phases in all nodules. In addition, PTLD was reported in other cases, several of which were characterized by the presence of PTLD after renal transplantation. However, none implemented CEUS to characterize intrahepatic lesions after HSCT.^[22]^ CEUS of PTLD after renal transplantation was similar in appearance, with peripheral to central enhancement followed by low enhancement. Because of the lack of similar cases, we will continue to collect cases and analyze the CEUS of intrahepatic PTLD after HSCT to provide valuable information for clinical diagnosis and treatment. The limitations of this case include the lack of a subsequent ultrasound after the condition of the patient improved and the unknown influence of drug intervention on the condition. Future research should focus on the clinical application and follow-up of therapeutic efficacy.

Small lesions of PTLD can be detected early via ultrasound. CEUS has fewer side effects, facilitates real-time observation, is less invasive, and can assist in evaluating tumor properties. Thus, US and CEUS are of great significance in the detection and diagnosis of PTLD after HSCT.

## Acknowledgments

I thank the other authors for their help with CEUS, and I want to express my appreciation to the other authors for their suggestions and guidance on scientific thinking. I would like to thank Editage (www.editage.com) for English language editing. My deepest gratitude goes to the reviewers for their serious work and pertinent advice.

## Author contributions

**Conceptualization:** Jianchun Li, Li Wang, Ligang Wang.

**Investigation:** Weinan Chen, Ailin Cui, Yang Liu.

**Supervision:** Jianchun Li, Xiaoming Fan, Yanming Zhang.

**Writing – original draft:** Weinan Chen.

**Writing – review & editing:** Weinan Chen, Yang Liu.

## Abbreviations

Abbreviations: CE-CT = contrast-enhanced computed tomography, CEUS = contrast-enhanced ultrasonography, CT = computed tomography, HSCT = hematopoietic stem cell transplantation, PTLD = post-transplant lymphoproliferative disease, US = ultrasonography.
